# The sensor histidine kinase PhcS participates in the regulation of quorum sensing-dependent virulence genes in *Ralstonia pseudosolanacearum* strain OE1-1

**DOI:** 10.1128/spectrum.00059-25

**Published:** 2025-03-04

**Authors:** Wakana Senuma, Masayuki Tsuzuki, Chika Takemura, Yuki Terazawa, Akinori Kiba, Kouhei Ohnishi, Kenji Kai, Yasufumi Hikichi

**Affiliations:** 1 Faculty of Agriculture and Marine Science, Kochi University, Nankoku, Japan; 2 Graduate School of Agriculture, Osaka Metropolitan University, Sakai, Japan; Lindsey Price Burbank, USDA-ARS San Joaquin Valley Agricultural Sciences Center, Parlier, California, USA

**Keywords:** *Ralstonia pseudosolanacearum*, PhcK, PhcS, quorum sensing, virulence

## Abstract

*Ralstonia pseudosolanacearum* strain OE1-1 secretes methyl 3-hydroxymyristate (3-OH MAME) as a quorum-sensing (QS) signal. Strain OE1-1 senses the chemical by the sensor histidine kinase PhcS, leading to the activation of the LysR family transcriptional regulator PhcA. The activated PhcA controls the expression of QS-dependent genes responsible for QS-regulated phenotypes including virulence. The autophosphorylation of the histidine at amino acid position 230 (H230-PhcS) in PhcS following the 3-OH MAME sensing is required for the PhcA activation. The alternative sensor histidine kinase PhcK is involved in the regulation of *phcA*, which is independent of 3-OH MAME sensing. Furthermore, the H230Q-PhcS substitution of H230-PhcS with glutamine significantly decreases *phcA* expression. However, how PhcK and PhcS regulate *phcA* expression remains unclear. To elucidate the mechanisms of the *phcA* regulation, we generated a *phcK* mutant with the H205Q-PhcK substitution of autophosphorylated histidine at amino acid position 205 of PhcK with glutamine. A transcriptome analysis using quantitative real-time polymerase chain reaction assay and RNA sequencing showed that the H230Q-PhcS substitution, but not the H205Q-PhcK substitution, significantly decreased the expression level of *phcA*. The H230Q-PhcS substitution led to significant changes in the expression levels of QS-dependent genes and a loss of virulence, similar to *phcA* or *phcK* deletion. It is thus thought that PhcS participates in not only the 3-OH MAME sensing-independently PhcK-mediated regulation of *phcA* but also the PhcA activation following 3-OH MAME sensing. Both functions of PhcS are significantly influenced by the autophosphorylation of H230-PhcS.

**IMPORTANCE:**

The soil-borne *Ralstonia solanacearum* species complex (RSSC) infects more than 300 plant species in over 50 families, including solanaceous plants, causing the devastating wilt disease that substantially decreases agricultural production worldwide. The cell density-dependent gene regulation system, QS, is required for RSSC virulence and involves two signaling pathways for the induction and activation of PhcA, which is the master transcriptional regulator in QS. In the present study, we describe the contribution of sensor histidine kinase PhcS to the PhcA induction, along with the alternative sensor kinase PhcK, independently of the sensing of QS signal methyl 3-hydroxymyristate in a phylotype I strain of RSSC, *R. pseudosolanacearum* strain OE1-1. This study further expands our knowledge of multiple networks, suggesting that several PhcS-mediated two-component systems are likely necessary for RSSC QS and virulence.

## INTRODUCTION

The cell density-dependent gene-regulating system, quorum sensing (QS), is a widely conserved bacterial cell−cell communication mechanism that coordinates numerous community activities. Bacteria secrete diffusible cell−cell signals (QS signals) and sense their surrounding concentrations to recognize their own populations. This leads to an activation of QS for the synchronous control of the expression of genes beneficial for vigorous replication, adaptation to environmental conditions, and virulence (1–4).

The soil-borne gram-negative β-proteobacterium *Ralstonia solanacearum* species complex (RSSC) infects more than 300 plant species in over 50 families, including solanaceous plants, causing the devastating wilt disease that substantially decreases agricultural production in the tropics, subtropics, and other regions with warm conditions (5). The RSSC is composed of four phylotypes, I–IV, and is assigned to three distinct species: *Ralstonia pseudosolanacearum* (phylotypes I and III), *R. solanacearum* (phylotype II), and *Ralstonia syzygii* (phylotype IV) (6).

During the infection of tomato roots, an RSSC phylotype I strain, *Ralstonia pseudosolanacearum* strain OE1-1 (7), first attaches to the epidermal surface in the root elongation zone and then colonizes the cortex surface to activate QS (8, 9). This leads to the induction of plant cell wall-degrading enzymes such as cellobiohydrolase (10), endoglucanase (11), and pectin methylesterase (12), which are secreted through type II secretion machinery (13) and degrade the cell walls of cortical cells (8, 9). The strain OE1-1 then infects the cell wall-denatured cortical cells to form mushroom-shaped biofilms, which are responsible for the virulence of strain OE1-1 on tomato plants. The formation of mushroom-shaped biofilms leads to further infection of the strain OE1-1 in xylem vessels. Therefore, QS is required for virulence of strain OE1-1 (9, 14).

Each RSSC strain secretes and senses either methyl 3-hydroxymyristate (3-OH MAME) or methyl 3-hydroxypalmitate as a QS signal (15–18). RSSC strains synthesize the QS signal by the methyltransferase PhcB and sense the chemical leads to the activation of the LysR family transcriptional regulator, PhcA, which controls the expression of QS-dependent genes responsible for QS-regulated phenotypes, including virulence (14, 19, 20). QS is conserved in all RSSC strains and is thus a required system for RSSC virulence. The phylogenetic study using amino acid sequences of PhcB and PhcS shows that RSSC strains are divided into two groups by their QS signal types, independently of their phylotypes (16–18). The ancestor prior to diversification of phylotypes could thus diverge into two types of QS signal production and sensing systems.

During the QS-active state at the higher bacterial density over the threshold, strain OE1-1 induces the production of the aryl-furanone secondary metabolite, ralfuranone (21–23); the major exopolysaccharide, EPS I (19, 20); a lectin LecM encoded by *lecM* (24, 25); and a plant cell wall-degrading enzyme, β-1,4-cellobiohydrolase (CbhA) encoded by *cbhA* (10). Notably, these chemical compounds are involved in the virulence of strain OE1-1 but also affect the expression level of QS-dependent genes (26–29).

**Fig 1:**
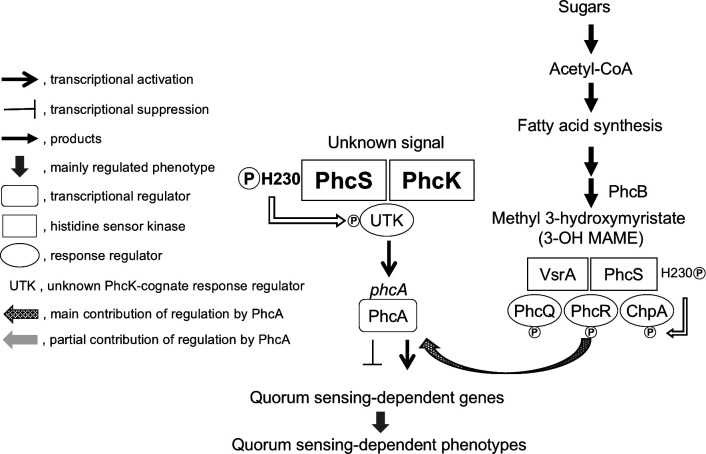
Predicted quorum-sensing signaling pathways in *Ralstonia pseudosolanacearum* strain OE1-1, which produces the QS signal, methyl 3-hydroxymyristate (3-OH MAME) synthesized by the methyltransferase PhcB. The strain OE1-1 senses 3-OH MAME through the sensor histidine kinases PhcS and VsrA. This leads to the autophosphorylation of the histidine at amino acid position 230 of PhcS (His-PhcS230). The phosphoryl group is transferred to the receiver domain of the cognate response regulators ChpA, PhcQ, and PhcR; ChpA and PhcQ contribute substantially to the regulation of QS-dependent genes via the LysR family transcriptional regulator PhcA, whereas PhcR makes a smaller contribution. The sensor histidine kinases PhcK and PhcS mediate the regulation of *phcA* independently of the sensing of 3-OH MAME. Autophosphorylated His-PhcS230 is also involved in the *phcA* regulation. In the active QS state, PhcA regulates the expression of the QS-dependent genes responsible for virulence.

In strain OE1-1, which produces 3-OH MAME as the QS signal (16–18), QS involves two signaling pathways (PhcA activation pathway and PhcA induction pathway) (Fig. 1) (9). In the PhcA activation pathway, the sensor histidine kinase PhcS is involved in the sensing of 3-OH MAME, along with the alternative sensor, histidine kinase VsrA, resulting in the autophosphorylation of the histidine at amino acid position 230 of PhcS (H230-PhcS) (30). This may induce the phosphorylation of the cognate response regulators PhcR, PhcQ, and ChpA, which are intracellular soluble regulators containing a receiver domain lacking a DNA-binding site, leading to the activation of PhcA (9, 31, 32). In the PhcA induction pathway, the sensor histidine kinase PhcK, which is involved in the sensing of unknown signals independently of bacterial density, is required for the full expression of *phcA* (9, 33). Additionally, the substitution (H230Q-PhcS) of H230-PhcS with glutamine leads to a significant decrease in *phcA* expression (33). However, how PhcK and PhcS regulate *phcA* expression remains unclear.

An analysis of the PhcK amino acid sequence deduced on the basis of the strain OE1-1 genome using the kinasephos2 algorithm (34) confirmed that histidine at amino acid position 205 of PhcK (H205-PhcK) is autophosphorylated (Fig. S1). It is thought that the phosphoryl group is transferred from the H205-PhcK to the receiver domain of the unknown cognate response regulator of PhcK, and the phosphorylated response regulator is involved in *phcA* regulation. In this study, to comprehensively analyze the effects of the H205Q-PhcK substitution of H205-PhcK with glutamine or H230Q-PhcS substitution on QS, we first created a *phcK* mutant (PhcK-H205Q) with the H205Q-PhcK substitution (Table 1). We then analyzed the expression level of *phcA* in *R. pseudosolanacearum* strains through a quantitative real-time polymerase chain reaction (qRT-PCR) assay. Furthermore, we performed transcriptome analyses of the *R. pseudosolanacearum* strains with RNA sequencing (RNA-seq). In addition, we assayed the productivity of major exopolysaccharide EPS I, which is a QS-regulated phenotype, as well as virulence on tomato of the PhcS-H230Q mutant with the H230Q-PhcS substitution with glutamine and PhcK-H205Q, as well as the *phcS*- and *phcK*-deletion mutants.

**TABLE 1:** Strains and plasmids used in this study

	Relevant characteristics	Source
Plasmids		
pK18mobsacB	Km^r^, *oriT* (RP4), *sacB*, *lacZα*	35
pPhcK-H205Q	pK18mobsacB derivative carrying a 1.2 kbp DNA fragment for H205Q substitution of PhcS, Km^r^	This study
pphcS-comp	pUC18-mini-Tn*7*T-Gm derivative carrying 2 kbp DNA fragment including the promoter of *phcBSR* operon and *phcS* for *phcS*-comp, Gm^r^	This study
*Escherichia coli* strain		
DH5α	*recA1 endA1 gyrA96 thi-1* *hsdR17supE44* Δ(*lac*)*U169* (Φ*80lac Δ*M15)	Takara Bio
*R. solanacearum* strains		
OE1-1	Wild-type strain, phylotype I, race 1, biovar 4	7
Δ*phcA*	*phcA*-deletion mutant of OE1-1	24
Δ*phcK*	*phcK*-deletion mutant of OE1-1	33
PhcK-H205Q	*phcK* mutant with H205Q substitution of OE1-1	This study
PhcS-H230Q	*phcS* mutant with H230Q substitution of OE1-1	30
Δ*phcS*	*phcS*-deletion mutant of OE1-1	30
*phcK*-comp	Transformant of Δ*phcK* with native *phcK*	33
*phcS*-comp	Transformant of Δ*phcS* with native *phcS*	This study

## RESULTS

### *phcA* expression in the PhcS-H230Q and PhcK-H205Q as well as the *phcS*- and *phcK*-deletion mutants

**Fig 2:**
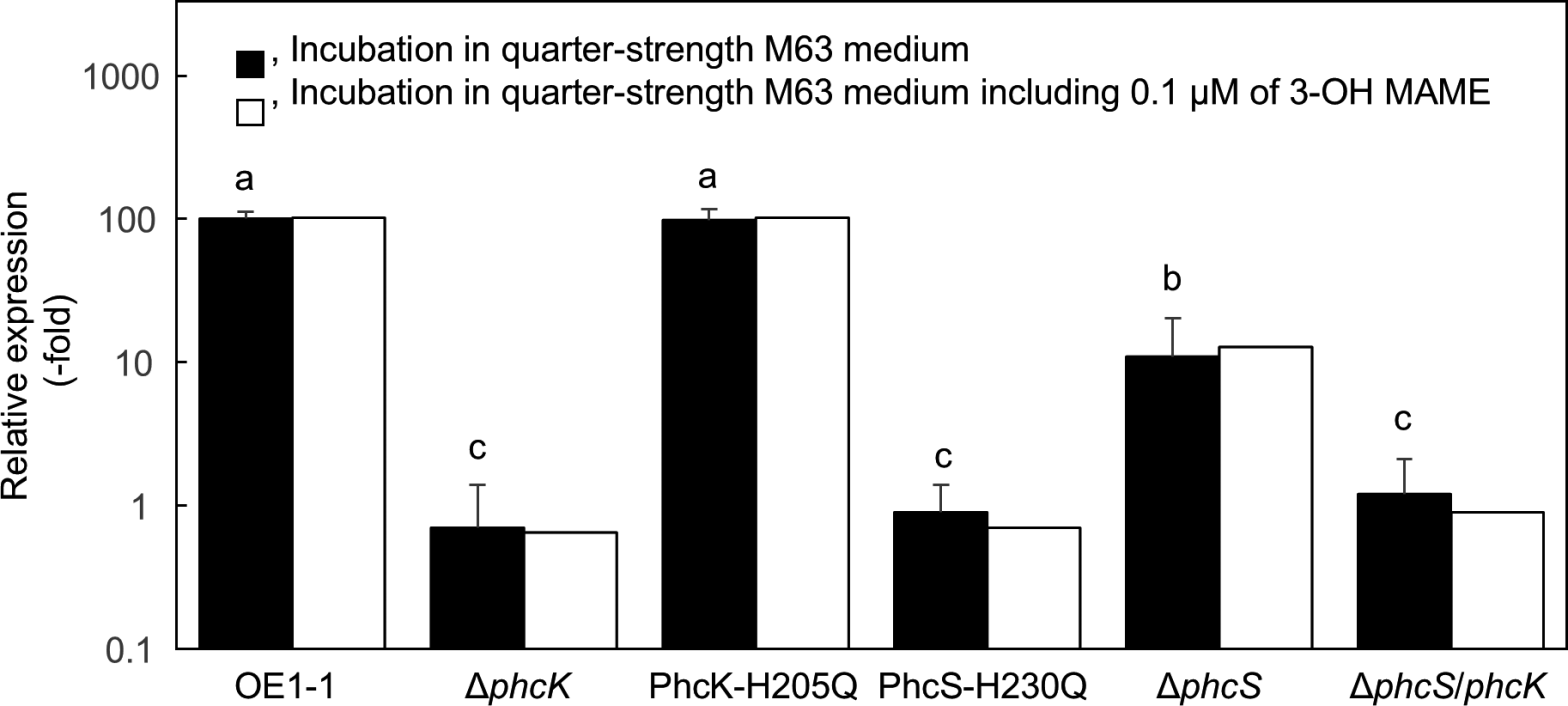
The quantitative real-time polymerase chain reaction analysis on *phcA* expression level in *Ralstonia pseudosolanacearum* strain OE1-1 and in several mutants (*phcA* deletion [Δ*phcA*], *phcS* deletion [Δ*phcS*], *phcK* deletion [Δ*phcK*], *phcK* mutant with the histidine at amino acid position 205 of PhcK substituted with glutamine [PhcK-H205Q], and *phcS* mutant with the histidine at amino acid position 230 of PhcS substituted with glutamine [PhcS-H230Q]) grown in quarter-strength M63 medium or quarter-strength M63 medium containing 0.1 µM methyl 3-hydroxymyristate (3-OH MAME). The *phcA* expression level was normalized against the *rpoD* expression level. Experiments were conducted using five biological replicates. Bars indicate standard errors. Means were analyzed for significant differences between *R. pseudosolanacearum* strains by an analysis of variance (ANOVA) followed by Tukey–Kramer’s honestly significant difference test. Statistically significant differences are indicated by different lowercase letters (*P* < 0.05). To assess the influence of exogenously applied 3-OH MAME, the means were analyzed for significant differences between the strains grown in quarter-strength M63 medium with and without 0.1 µM 3-OH MAME by an ANOVA followed by Student’s *t*-test (*P* < 0.05).

We first analysed the expression level of *phcA* in *R. pseudosolanacearum* strains incubated in quarter-strength M63 medium, which we have used to analyse the QS signalling pathway as a model incubation system (9), through a qRT-PCR assay. The expression level of *phcA* was significantly lower in the *phcS* deletion mutant (Δ*phcS*, Table 1), compared to that in the strain OE1-1 (*P* < 0.05; Fig. 2). However, the level was significantly greater in the Δ*phcS* than in the *phcK* deletion mutant (Δ*phcK*, Table 1). The *phcS* mutant (PhcS-H230Q, Table 1) with the H230Q-PhcS substitution, but not the PhcK-H205Q, showed significantly decreased *phcA* expression level, similar to the Δ*phcK*. The rank-order of *phcA* expression level was as follows: OE1-1 ≈ PhcK-H205Q > Δ*phcS* > PhcS-H230Q ≈ Δ*phcK*.

The exogenous application with 3-OH MAME at a concentration of 0.1 µM leads to an enhanced PhcA activation pathway, increasing the QS-inducible production of major exopolysaccharide EPS I (31). Though PhcK is required for the full expression of *phcA*, exogenous 3-OH MAME application does not affect the expression level of *phcA* in the strain OE1-1, indicating that the expression level of *phcA* is regulated independently of 3-OH MAME sensing (33). We then analyzed the expression level of *phcA* in *R. pseudosolanacearum* strains when grown in quarter-strength M63 medium containing 0.1 µM 3-OH MAME. The qRT-PCR data showed that the presence of 3-OH MAME in the growth medium did not affect the expression level of *phcA* in strain OE1-1 or the mutants (Fig. 2).

### Transcriptome analysis of the PhcS-H230Q and PhcK-H205Q as well as the *phcS*- and *phcK*-deletion mutants with RNA-seq

To comprehensively analyze the effects of the H205Q-PhcK or H230Q-PhcS substitution on gene regulation, we performed transcriptome analyses of *R. pseudosolanacearum* strains with RNA-seq, and 4,327 protein-coding transcripts were identified by the mapping of strain OE1-1 RNA-seq reads to the GMI1000 genome (36) (Table S1).

The comparison with OE1-1 revealed a lack of significant change in the expression levels of QS-related *phcB*, *phcK*, *phcS*, *vsrA*, *phcQ*, *phcR*, and *chpA* in the PhcK-H205Q and PhcS-H230Q mutants. However, the H230Q-PhcS substitution, but not the H205Q-PhcK substitution, significantly decreased the expression level of *phcA*, similar to the effect of deleting *phcK* (Table S2).

**Fig 3:**
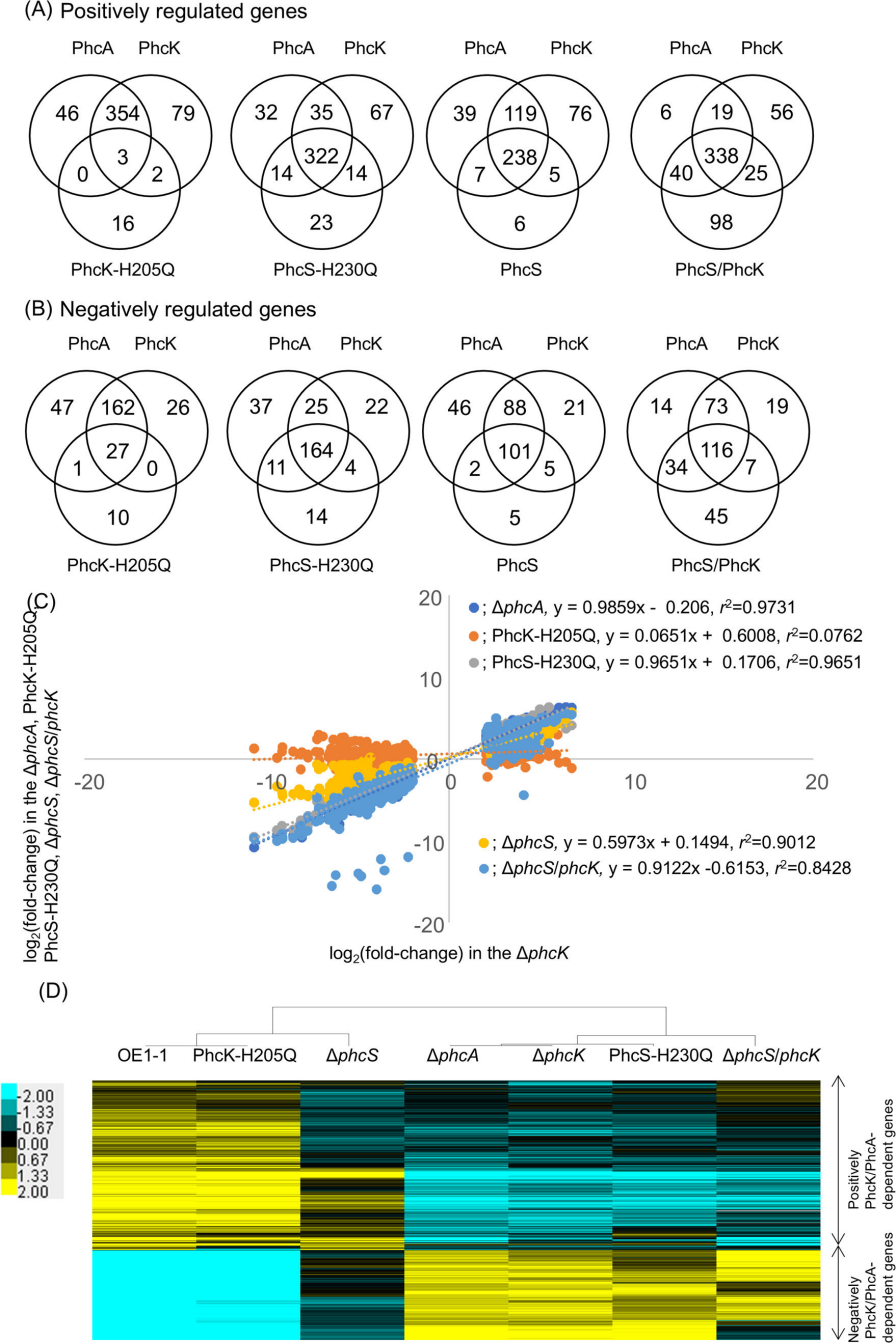
RNA-sequencing transcriptome analysis of *Ralstonia pseudosolanacearum* strain OE1-1 and several mutants (*phcA* deletion [Δ*phcA*], *phcS* deletion [Δ*phcS*], *phcK* deletion [Δ*phcK*], *phcK* mutant with the histidine at amino acid position 205 of PhcK substituted with glutamine [PhcK-H205Q], and *phcS* mutant with the histidine at amino acid position 230 of PhcS substituted with glutamine [PhcS-H230Q]). The results of the analysis of the genes with log_2_(fold change) ≤−2 (A) or ≥2 (B) in Δ*phcA*, Δ*phcS*, Δ*phcK*, PhcS-H230Q, and PhcK-H205Q relative to their expression levels in strain OE1-1 (*q* value <0.05) are presented. (C) Correlations among the expression levels of PhcK/PhcA-dependent genes revealed by the comparison of Δ*phcK* with Δ*phcS*, Δ*phcA*, PhcS-H230Q, or PhcK-H205Q. (D) Hierarchical clustering of the relative expression of PhcK/PhcA-dependent genes in *R. pseudosolanacearum* strains.

Comparison with the expression levels of protein-coding transcripts in strain OE1-1 showed that 256 genes (positively PhcS-dependent genes) had significantly downregulated expression levels in Δ*phcS*, whereas 133 genes (negatively PhcS-dependent genes) had significantly upregulated expression levels in Δ*phcS* (Fig. 3A and B; Table S3). In PhcS-H230Q, the expression levels of 373 genes (positively H230Q-dependent genes) were significantly downregulated, which were in contrast to the significantly upregulated expression levels of 193 genes (negatively H230Q-dependent genes) (Fig. 3A and B; Table S4). Among the positively and negatively PhcS-dependent genes, 239 and 98 were identified as positively and negatively H230Q-dependent genes, respectively.

The comparison with strain OE1-1 detected 436 positively PhcK-dependent genes and 215 negatively PhcK-dependent genes in the Δ*phcK*, with significantly downregulated and upregulated expression levels, respectively (Fig. 3A and B; Table S5). The His-PhcK205-Gln substitution significantly decreased the expression of 21 genes (positively H205Q-dependent genes) but significantly increased the expression of 38 genes (negatively H205Q-dependent genes) (Fig. 3A and B; Table S6). Among the positively and negatively H205Q-dependent genes, 5 and 27 were included among the positively and negatively PhcK-dependent genes, respectively.

Of the positively and negatively PhcK-dependent genes, 357 and 189 genes (positively and negatively PhcK/PhcA-dependent genes) had significantly downregulated and upregulated expression levels in the *phcA*-deletion mutant (Δ*phcA*), respectively (Fig. 3A and B; Table S7). Thus, the full expression of *phcA* dependent on PhcK appears to contribute to the regulation of these genes.

Of the positively PhcK/PhcA-dependent genes and negatively PhcK/PhcA-dependent genes, 3 and 27 genes were identified as positively H205Q-dependent genes and negatively H205Q-dependent genes, respectively (Fig. 3A and B). Moreover, 238 positively PhcK/PhcA-dependent genes and 101 negatively PhcK/PhcA-dependent genes were included among the positively PhcS-dependent genes and negatively PhcS-dependent genes, respectively. Furthermore, 322 and 164 positively and negatively PhcK/PhcA-dependent genes were also designated as positively and negatively H230Q-dependent genes, respectively. In addition, 338 and 116 positively and negatively PhcK/PhcA-dependent genes were also considered to be positively and negatively PhcK/PhcA-dependent genes, respectively.

The transcript levels of PhcK/PhcA-dependent genes were positively correlated between Δ*phcA* and Δ*phcK* [*y*, log_2_(fold change) in Δ*phcK*; *x*, log_2_(fold change) in Δ*phcA*; *y* = 0.9871*x* − 0.0213, *r*^2^ = 0.9731; Fig. 3C]. Similarly, the transcript levels of PhcK/PhcA-dependent genes were positively correlated between Δ*phcA* and Δ*phcS* [*y*, log_2_(fold change) in Δ*phcS*; *x*, log_2_(fold change) in Δ*phcA*; *y* = 0.599*x* + 0.1526, *r*^2^ = 0.9079]. Furthermore, the transcript levels of PhcK/PhcA-dependent genes were highly positively correlated between Δ*phcA* and PhcS-H230Q [*y*, log_2_(fold change) in PhcS-H230Q; *x*, log_2_(fold change) in Δ*phcA*; *y* = 0.9271*x* + 0.1692, *r*^2^ = 0.9636]. In contrast, the correlation between the transcript levels of PhcK/PhcA-dependent genes between Δ*phcA* and PhcK-H205Q was relatively low [*y*, log_2_(fold change) in PhcK-H205Q; *x*, log_2_(fold change) in Δ*phcA*; *y* = 0.068*x* + 0.6052, *r*^2^ = 0.0829].

The dendrogram for the hierarchical clustering of *R. pseudosolanacearum* strains was created based on their relative expression levels normalized against the expression of PhcK/PhcA-dependent genes, revealing that PhcS-H230Q was clustered with Δ*phcK* and Δ*phcA*, whereas PhcK-H205Q was grouped with strain OE1-1 and Δ*phcS* (Fig. 3D).

### Influence of the H230Q-PhcS230 but not H205Q-PhcK substitution on QS-regulated EPS I productivity

**Fig 4:**
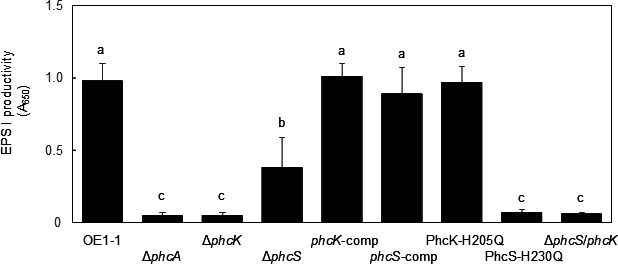
Production of major exopolysaccharide (EPS I) in *Ralstonia pseudosolanacearum* strain OE1-1 and several mutants (*phcA* deletion [Δ*phcA*], *phcS* deletion [Δ*phcS*], *phcK* deletion [Δ*phcK*], complemented Δ*phcS* with native *phcS* [*phcS*-comp], complemented Δ*phcK* with native *phcK* [*phcK*-comp], *phcK* mutant with the histidine at amino acid position 205 of PhcK substituted with glutamine [PhcK-H205Q], and *phcS* mutant with the histidine at amino acid position 230 of PhcS substituted with glutamine [PhcS-H230Q]). The experiment was repeated seven times. Bars indicate standard errors. Means were analyzed for significant differences between *R. pseudosolanacearum* strains by an analysis of variance followed by Tukey–Kramer’s honestly significant difference test. Statistically significant differences are indicated by different lowercase letters (*P* < 0.05).

Exopolysaccharides are the main virulence factors of RSSC, and their production is positively regulated by QS (19, 20). Moreover, the major exopolysaccharide EPS I is associated with the feedback regulation of QS (27). To analyze the effects of the H205Q-PhcK or H230Q-PhcS230 substitution on QS-regulated phenotypes, EPS I production in *R. pseudosolanacearum* strains was quantitatively analyzed. The Δ*phcS* exhibited significantly less EPS I production, compared with strain OE1-1 (*P* < 0.05, Fig. 4). However, EPS I production was significantly greater in Δ*phcS* than in Δ*phcA* and Δ*phcK*. The H230Q-PhcS substitution, but not the H205Q-PhcK substitution, significantly decreased EPS I production, similar to the effects of deleting *phcK* or *phcA*. The complemented Δ*phcS* with native *phcS* (*phcS*-comp, Table 1) showed similar EPS I productivity to the complemented Δ*phcK* with native *phcK* (*phcK*-comp, Table 1). The rank order of EPS I production was as follows: OE1-1 ≈ *phcK*-comp ≈ *phcS*-comp ≈ PhcK-H205Q > Δ*phcS* >PhcS-H230Q ≈ Δ*phcK* ≈ Δ*phcA*.

### Loss in virulence of the PhcS-H230Q but not PhcK-H205Q on tomato plants

**Fig 5:**
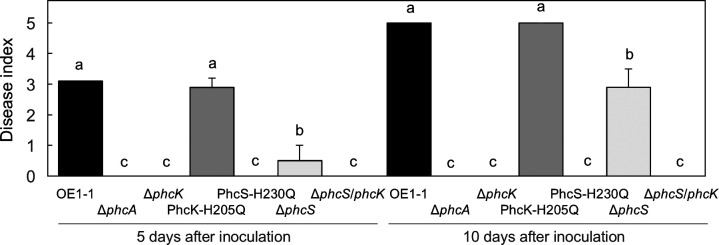
Bacterial wilt on tomato plants inoculated with *Ralstonia pseudosolanacearum* strain OE1-1 and several mutants (*phcA* deletion [Δ*phcA*], *phcS* deletion [Δ*phcS*], *phcK* deletion [Δ*phcK*], *phcK* mutant with the histidine at amino acid position 205 of PhcK substituted with glutamine [PhcK-H205Q], and *phcS* mutant with the histidine at amino acid position 230 of PhcS substituted with glutamine [PhcS-H230Q]). Plants were rated according to the following disease index scale: 0, no wilting; 1, 1%–25% wilting; 2, 26%–50% wilting; 3, 51%–75% wilting; 4, 76%–99% wilting; and 5, dead. For each bacterial strain, three independent groups were tested, with 12 technical replicates per group. We analyzed means for significant differences between *R. pseudosolanacearum* strains by analysis of variance followed based on Tukey–Kramer’s honestly significant difference test (*P* < 0.05).

The Δ*phcK* loses its virulence, similar to the Δ*phcA* (33). To analyze the effects of the H230Q-PhcS and H205Q-PhcK substitutions on virulence, we assayed wilt symptoms on tomato plants inoculated with *R. pseudosolanacearum* strains. The plants inoculated with PhcK-H205Q had detectable wilt symptoms at 5 days after inoculation and were eventually dead by 10 days after inoculation, similar to the plants inoculated with OE1-1 (Fig. 5). The plants inoculated with Δ*phcS* had weaker wilt symptoms than the plants inoculated with OE1-1 (*P* < 0.05). However, the plants inoculated with PhcS-H230Q were not wilted at all, similar to the plants inoculated with the Δ*phcK* or Δ*phcA*. The rank order of virulence was as follows: OE1-1 ≈ PhcK-H205Q > Δ*phcS* > PhcS-H230Q ≈ Δ*phcK* ≈ Δ*phcA* ≈ avirulent strain. The rank order of virulence among *R. pseudosolanacearum* strains is thus positively connected to the rank order of EPS I production as well as *phcA* expression level.

## DISCUSSION

The QS is the central signaling pathway for virulence of RSSC (9, 14, 19, 20). There are multiple additional regulatory proteins that play supporting roles in RSSC virulence (20, 37). The VsrAD two-component system, which consists of a sensor histidine kinase VsrA and a cognate response regulator VsrD, controls multiple virulence-related traits independently of the QS (19, 20, 38, 39). The VsrAD is required for bioﬁlm formation, tolerance of both cold temperatures and hydrogen peroxide, and acyl-homoserine lactone production. The VsrAD is involved in the positive control of the expression of *cbhA* and the production of EPS I and ralfuranone. On the other hand, VsrAD negatively controls swimming. Although many of the VsrAD-regulated traits are also controlled by PhcA, the multiple phenotypic and regulatory differences indicate that these are separate regulons (19, 20). Our previous study demonstrated that in the PhcA activation pathway, the involvement of PhcS in the sensing of QS signal 3-OH MAME, along with VsrA, leads to the activation of PhcA regulating QS-dependent genes responsible for significant changes in QS-regulated phenotypes of strain OE1-1 and leading to its virulence (30). It is thus thought that multiple networks involving PhcS- and VsrA-mediated two-component systems are likely necessary for RSSC QS and virulence. Via the PhcA induction pathway, the *phcA* expression was induced independently of 3-OH MAME sensing as shown in Fig. 2, indicating that the regulation of *phcA* is independently of bacterial density of the strain OE1-1. PhcK greatly contributes to the regulation of *phcA* (33). Intriguingly, the results of this study indicate that PhcS also contributes to the regulation of *phcA*, along with PhcK, independently of 3-OH MAME sensing. In addition, the deletion of *cbhA*, which encodes β-1,4-endoglucanase and whose expression is induced by PhcA in the QS-active state, significantly decreased the expression level of *phcA*, thereby significantly reducing virulence (29). These pieces of evidence indicate that the full expression of *phcA* significantly contributes to the regulation of QS-dependent genes and virulence. Therefore, PhcS is the vital sensor histidine kinase for both the PhcA induction and PhcA activation, respectively, along with PhcK and VsrA, leading to QS activation. Multiple networks involving several PhcS-mediated two-component systems are likely necessary for RSSC QS and virulence.

The two-component signal transduction system generally comprises an inner membrane-bound sensor histidine kinase and a cytosolic response regulator. The typical sensor histidine kinases have a variable N-terminal sensor domain to detect a specific signal, a C-terminal containing a conserved transmitter domain to hydrolyze ATP, and a side chain with a histidine residue autophosphorylated following the detection of a particular signal. The phosphoryl group is then transferred to the receiver domain of the cognate response regulator (40–42). In the PhcA activation pathway, the autophosphorylation of the H230-PhcS but not the histidine at amino acid position 258 of VsrA is essential for the phosphorylation of the cognate response regulators PhcR, PhcQ, and ChpA (31, 32). Though the absence of both *phcS* and *vsrA* leads to significant changes in QS-regulated phenotypes, the absence of *vsrA* does not lead to changes in the QS-regulated phenotypes. Furthermore, the absence of *phcS* results in minor changes in the QS-regulated phenotypes, suggesting that the downstream cognate response regulators of the phosphorylation cascade in the absence of PhcS can still be phosphorylated by other kinases including VsrA. In the PhcA induction pathway, we previously demonstrated that PhcK is the essential sensor histidine kinase for the PhcA induction (33). However, results in our present study suggested that the phosphoryl group is transferred from phosphorylated H230-PhcS but not H205-PhcK to an unknown cognate response regulator (PhcK/PhcS-cognate response regulator) for the *phcA* regulation. PhcS is thus involved in both PhcA activation, which depends on 3-OH MAME sensing, and PhcA induction, which occurs independently of 3-OH MAME sensing. Phosphorylated H230-PhcS is required for both functions of PhcS. It is thus thought that PhcS can function in different manners in response to its cognate sensor histidine kinase. In general, sensor histidine kinases comprise amino acids that enable the formation of homodimers that facilitate autophosphorylation (43). However, a physiologically relevant heterodimerization of GacS and RetS has been confirmed in *Pseudomonas aeruginosa* (44). Furthermore, the bifunctional histidine kinase/phosphatase of the HWE family of *Sinorhizobium meliloti* (45) and the LovK-LovR two-component system of *Caulobacter crescentus* (46) have been reported. Because our results were based on the transcriptome analysis and QS-regulated phenotypes, including virulence of *R. pseudosolanacearum* strains, how PhcS functions in the PhcA activation pathway, which depends on 3-OH MAME sensing, and in the PhcA induction pathway, which occurs independently of 3-OH MAME sensing, respectively, along with VsrA and PhcK, remains unknown. To certify our results and elucidate these mechanisms, further *in vitro* experiments using puriﬁed sensor histidine kinases and phosphorylation assays may be required.

Results of our previous and present studies showed that the Δ*phcS* exhibited minor changes in the QS-regulated phenotypes and virulence (30). Moreover, the H205Q-PhcK substitution did not significantly affect the regulation of PhcK/PhcA-dependent genes and QS-regulated EPS I production as well as virulence. It is thus thought that in the absence of PhcS, an alternative sensor histidine kinase may contribute to the regulation of *phcA*, along with PhcK, with the phosphoryl group being transferred from the autophosphorylated histidine of the alternative sensor histidine kinase to the receiver domain of the PhcK/PhcS-cognate response regulator. In the present study, His-PhcS230 contributed to the full expression of *phcA* independently of the sensing of 3-OH MAME. According to the results of these studies, we propose the predicted quorum-sensing signaling pathways in the strain OE1-1 (Fig. 1). In the PhcA activation pathway, PhcS is involved in the 3-OH MAME sensing along with VsrA, leading to the autophosphorylation of His-PhcS230 (30). The phosphoryl group is transferred to the receiver domain of the cognate response regulators ChpA, PhcQ, and PhcR; ChpA and PhcQ contribute substantially to the regulation of QS-dependent genes via PhcA, whereas PhcR makes a smaller contribution (31, 32). PhcK and PhcS mediate the full expression of *phcA* independently of the sensing of 3-OH MAME. Additionally, autophosphorylated His-PhcS230 significantly contributes to the *phcA* regulation. In summary, these findings suggest that PhcS, PhcK, and VsrA are sensor histidine kinases that may function together as well as with other nonspecific sensor histidine kinases to regulate both the production and the activation of PhcA during the QS-active state. Multiple networks involving several PhcS-mediated two-component systems are likely necessary for RSSC QS and virulence.

The bacterial density-dependent gene regulation system QS is involved in the behavior of RSSC in host roots and is required for virulence of RSSC (14). QS-inducible and virulence-related metabolites, EPS I and ralfuranone, are separately involved in PhcA-dependent gene regulation (26, 27). During the activation stage of QS, the expression level of *lecM* encoding a lectin LecM, which is located in outer membranes, is significantly enhanced by PhcA (24). LecM in the planktonic stage at the lower bacterial concentration is involved in the attachment of strain OE1-1 on plant cell surfaces. During the activation stage of QS at the higher bacterial concentration, LecM is involved in the stability of extracellularly secreted 3-OH MAME (28). Furthermore, CbhA contributes to not only the invasion of strain OE1-1 into cortical cells and xylem vessels but also the full expression of *phcA* (29). Therefore, EPS I, ralfuranone, LecM, and CbhA participate in the feedback regulation of the QS. Furthermore, intracellular concentration of divalent iron greatly influences the regulation of PhcA-regulated genes. One of the siderophores, micacocidine, of strain OE1-1 is involved in the regulation of PhcA-regulated genes responsible for QS-regulated phenotypes, including virulence, as well as trivalent iron-scavenging activity (47). Furthermore, the 3-OH MAME analogs inhibit QS-regulated phenotypes and reduce the virulence of strain OE1-1 on tomato plants (48). On the other hand, it is reported that a *phcA* mutant growing in culture has a different transcriptional proﬁle from the same mutant during tomato infection (49, 50). Further analysis on the signaling pathways and regulation system of QS in RSSC with which host plants are infected is thus needed for the elucidation of virulence mechanisms of RSSC.

## MATERIALS AND METHODS

### Conditions for bacterial growth

All *R. pseudosolanacearum* strains listed in Table 1 were routinely grown in quarter-strength M63 medium [4 mM (NH_4_)_2_SO_4_, 0.02 M KH_2_PO_4_, 450 nM FeSO_4_·7H_2_O, 0.67 mM MgSO_4_, 0.02 M C_5_H_8_NO_4_Na·H_2_O] (pH 7.0) at 30°C. To incubate *Escherichia coli* strain DH5α (Table 1) and its transformants with plasmids (Table 1) at 37°C, Luria–Bertani medium (51) was used.

### Generation of the PhcK-H205Q mutant

To generate PhcK-H205Q, we first created the plasmid pPhcK-H205Q (Table 1), in which recombinant PCR fragments using primers (Table 2) were inserted into pK18mobsacB (52). pPhcK-H205Q was electroporated into competent cells of OE1-1 to select the kanamycin-sensitive and sucrose-resistant recombinant, PhcK-H205Q. Using the DNA sequencing data, we confirmed that the PhcK-H205Q mutant harbors mutated PhcK with the H205Q-PhcK substitution, but not native PhcK.

**TABLE 2:** Primers used in the creation of plasmid pPhcK-H205Q for the generation of the *phcK* mutant with the substitution of histidine at amino acid position 205 in PhcK with glutamine^*a*^*^,^*^*b*^

Plasmid	Primers	Nucleotide sequences
pPhcK-H205Q	H205Q-1-FW	5′-cggaattcGCCAACGTGTCGCTGCCTC-3′
	H205Q-1-RV	5′-CACCTGGGCCAGCGCGTT-3′
	H205Q-2-FW	5′-AACGCGCTGGCCCAGGTG-3′
	H205Q-2-RV	5′-cccaagcttGCCGGTTCATCGTCGGCAATC-3′

^*a*^ Lowercase letters denote restriction enzyme sites.

^*b*^ Underlined letters denote mutation sites.

### Generation of a *phcS*-deletion mutant transformed with native *phcS*

To create the plasmid pphcS-comp (Table 1) including the open reading frame of *phcS* fused with the promoter of the *phcBSR* operon, the fragments comp-S-1 and comp-S-2 were amplified by PCR from the genomic DNA of strain OE1-1 using the oligonucleotide primers listed in Table 3. The BamHI- and HindIII-digested fragment comp-S, amplified using comp-S-1 and comp-Q-2, was ligated into a BamHI- and HindIII-digested pUC18-mini-Tn*7*T-Gm vector (35) to produce pphcS-comp. This plasmid was electroporated into Δ*phcS* competent cells with a T7 transposase expression vector, pTNS2 (35). Finally, a gentamicin-resistant transformant, *phcS*-comp (Table 1), was selected.

**TABLE 3:** Primers used in the creation of plasmid pphcS-comp for the generation of the *phcS*-deletion mutant with the native *phcS^a^*

Plasmid	Primers	Nucleotide sequences
pphcS-comp	compS-1-FW	5′-agggcctgcgcgaaaaacag-3′
	compS-1-RV	5′-CGGCGATCATggtgcgaatttgccggagac-3′
	compS-2-FW	5′-caaattcgcaccATGATCGCCGCCAACTACCAG-3′
	compS-2-RV	5′-cccaagcttGCTCGCTCCTATTCCGCG-3′

^*a*^ Lowercase letters denote restriction enzyme sites.

### Generation of a *phcK*-deletion mutant transformed with native *phcK*

We used the gentamycin-resistant *phcK*-comp, which is the Δ*phcK* transformed with the pUC18-mini-Tn7T-Gm vector (35)-based plasmid containing the native *phcK* and its promoter (33).

### qRT-PCR

The expression level of *phcA* in strain OE1-1 and in the mutants was analyzed through a qRT-PCR assay. Total RNA was extracted using a High Pure RNA Isolation kit (Roche Diagnostics, Tokyo, Japan) from *R. pseudosolanacearum* strains which were grown in quarter-strength M63 medium until the optical density at 600 nm reached 0.3 as previously described (28). A qRT-PCR using gene-specific primers (Table 4) was completed with a SYBR GreenER qPCR Reagent system (Invitrogen, Tokyo, Japan) and a 7300 Real-Time PCR platform (Applied Biosystems, Foster City, CA, USA). The transcript level of *rpoD* was used as the internal standard to normalize all values. We did not observe any significant differences in the *rpoD* transcript level among the *R. pseudosolanacearum* strains. We used an analysis of variance (ANOVA) followed by Tukey–Kramer’s honestly significant difference test to analyze the means of five biological replicates for significant differences between *R. pseudosolanacearum* strains (*P* < 0.05).

**TABLE 4:** Primers used in the quantitative real-time polymerase chain reaction assays

Genes	Primers	Nucleotide sequences
*rpoD*	rpoD-FW	5′-ATCGTCGAGCGCAACATCCC-3′
	rpoD-RV	5′-AGATGGGAGTCGTCGTCGTCGTG-3′
*phcA*	phcA-FW5	5′-ATGCGTTCCAATGAGCTGGAC-3′
	phcA-RV5	5′-AGATCCTTCATCAGCGAGTTGAC-3′

### Transcriptome analysis based on RNA sequencing

The transcriptomes in *R. pseudosolanacearum* strains were analyzed through RNA sequencing as previously described (26–33, 47). Briefly, we extracted the total RNA from *R. pseudosolanacearum* strains and eliminated ribosomal RNA using a Ribo-Zero rRNA Removal kit (gram-negative bacteria) (Illumina, Madison, WI, USA). Bioengineering Lab Co. (Sagamihara, Japan) performed oriented, paired-end RNA sequencing (2 × 100 bp) on the Illumina HiSeq 2500 system (Illumina) and DNBSEQ-G400 system (MGI Tech, Shenzhen, China) and trimmed the generated reads with Cutadapt (53) and Trimmomatic (54) to map to the genome of *R. pseudosolanacearum* strain GMI1000 (36) with the TopHat program (55). The average of at least three biological replicates per strain was calculated.

Statistical analysis of the RNA-seq data was performed in the R environment (56). Genes with zero counts in at least one OE1-1 sample were excluded in the raw count data set. To normalize the RNA-seq read counts of the remaining genes, we used the function calcNormFactors (trimmed mean of *M* value normalization) in the package edgeR (57). To screen for genes with significant changes in transcription, the following thresholds were applied: *q* value <0.05 and |log_2_(fold change)| ≥2. We calculated the false discovery rate (*q* value) on the basis of the *P* values estimated by edgeR using the Benjamini–Hochberg method (58). We used Cluster version 3.0 (59) for the hierarchical clustering of all normalized mean transcript values on the basis of their relative transcript levels (counts per million) and TreeView (60) for the creation of heatmaps.

### EPS I productivity

We quantitatively analyzed the production of major exopolysaccharide EPS I in *R. pseudosolanacearum* strains grown on quarter-strength M63 medium solidified with 1.5% (wt/vol) agar using an enzyme-linked immunosorbent assay (Agdia, Elkhart, IN, USA) to measure the absorbance at 650 nm as previously described (24). We analyzed the means of seven biological replicates for significant differences between *R. pseudosolanacearum* strains using ANOVA followed by Tukey–Kramer’s honestly significant difference test (*P* < 0.05).

### Virulence assays

Eight-week-old tomato plants (*Solanum lycopersicum* cultivar Ohgata-Fukuju) were inoculated with *R. pseudosolanacearum* strains at 1.0 × 10^8^ colony-forming unit/mL by a root-dip method, and the plants with wilting symptoms were monitored daily as previously described (24): 0, no wilting; 1, 1%–25% wilting; 2, 26%–50% wilting; 3, 51%–75% wilting; 4, 76%–99%; and 5, dead. For each bacterial strain, three independent groups were tested, with 12 technical replicates per group. Means were analyzed for significant differences between *R. pseudosolanacearum* strains by analysis of variance followed by Tukey–Kramer’s honestly significant difference test (*P* < 0.05).

## Data Availability

The data that support the findings of this study are available from the corresponding author upon reasonable request. RNA-sequencing data are available in the National Center for Biotechnology Information Sequence Read Archive repository (https://www.ncbi.nlm.nih.gov/sra/) under accession codes DRR493625, DRR438005, DRR438006, DRR438007, and DRR493446 (wild type: OE1-1); DRR438008, DRR438009, DRR438010, DRR450853, DRR450854, and DRR493614 (∆phcA); DRR512175, DRR512176, and DRR512177 (∆phcK); DRR512178, DRR512179, and DRR512180 (∆phcS); DRR512187, DRR512188, and DRR512189 (PhcS-H230Q); and DRR512190, DRR512191, and DRR512192 (PhcK-H205Q).

## SUPPLEMENTAL MATERIAL

Fig S1 spectrum.00059-25-s0001.tif Domains in histidine sensor kinases, PhcK and PhcS, of *Ralstonia pseudosolanacearum* strain OE1-1 analyzed by the kinasephos2 algorithm (40) using deduced amino acid sequences.

Supplemental material spectrum.00059-25-s0002.docx Fig. S1 legend.

Table S1 spectrum.00059-25-s0003.xlsx RNA-sequencing data for transcripts of genes in *Ralstonia pseudosolanacearum* strain OE1-1, and phcA deletion (ΔphcA), phcK deletion (ΔphcK) and phcS deletion (ΔphcS) mutants, phcK mutant with the substitution of histidine at amino acid position 205 in PhcK to glutamine (PhcK-H205Q), phcS mutant with the substitution of histidine at amino acid position 230 to glutamate (PhcS-H230Q), grown in quarter-strength M63 medium, and predicted function of proteins encoded by the genes.

Table S2 spectrum.00059-25-s0004.xlsx Predicted quorum sensing signaling pathways in *Ralstonia pseudosolanacearum* strain OE1-1.

Table S3 spectrum.00059-25-s0005.xlsx RNA-sequencing data for transcripts of PhcS-dependent genes in *Ralstonia pseudosolanacearum* strain OE1-1, and phcA deletion (ΔphcA), phcK deletion (ΔphcK) and phcS deletion (ΔphcS) mutants, phcK mutant with the substitution of histidine at amino acid position 205 in PhcK to glutamine (PhcK-H205Q), phcS mutant with the substitution of histidine at amino acid position 230 to glutamate (PhcS-H230Q), grown in quarter-strength M63 medium, and predicted the function of proteins encoded by the genes.

Table S4 spectrum.00059-25-s0006.xlsx RNA-sequencing data for transcripts of H230Q-dependent genes in *Ralstonia pseudosolanacearum* strain OE1-1, and phcA deletion (ΔphcA), phcK deletion (ΔphcK) and phcS deletion (ΔphcS) mutants, phcK mutant with the substitution of histidine at amino acid position 205 in PhcK to glutamine (PhcK-H205Q), phcS mutant with the substitution of histidine at amino acid position 230 to glutamate (PhcS-H230Q), grown in quarter-strength M63 medium, and predicted function of proteins encoded by the genes.

Table S5 spectrum.00059-25-s0007.xlsx RNA-sequencing data for PhcK-dependent genes in *Ralstonia pseudosolanacearum* strain OE1-1, and phcA deletion (ΔphcA), phcK deletion (ΔphcK) and phcS deletion (ΔphcS) mutants, phcK mutant with the substitution of histidine at amino acid position 205 in PhcK to glutamine (PhcK-H205Q), phcS mutant with the substitution of histidine at amino acid position 230 to glutamate (PhcS-H230Q), grown in quarter-strength M63 medium, and predicted function of proteins encoded by the genes.

Table S6 spectrum.00059-25-s0008.xlsx RNA-sequencing data for transcripts of H205Q-dependent genes in *Ralstonia pseudosolanacearum* strain OE1-1, and phcA deletion (ΔphcA), phcK deletion (ΔphcK) and phcS deletion (ΔphcS) mutants, phcK mutant with the substitution of histidine at amino acid position 205 in PhcK to glutamine (PhcK-H205Q), phcS mutant with the substitution of histidine at amino acid position 230 to glutamate (PhcS-H230Q), grown in quarter-strength M63 medium, and predicted function of proteins encoded by the genes.

Table S7 spectrum.00059-25-s0009.xlsx RNA-sequencing data for transcripts of PhcK/PhcA-dependent genes in *Ralstonia pseudosolanacearum* strain OE1-1, and phcA deletion (ΔphcA), phcK deletion (ΔphcK) and phcS deletion (ΔphcS) mutants, phcK mutant with the substitution of histidine at amino acid position 205 in PhcK to glutamine (PhcK-H205Q), phcS mutant with the substitution of histidine at amino acid position 230 to glutamate (PhcS-H230Q), grown in quarter-strength M63 medium, and predicted function of proteins encoded by the genes.

ASM does not own the copyrights to Supplemental Material that may be linked to, or accessed through, an article. The authors have granted ASM a non-exclusive, world-wide license to publish the Supplemental Material files. Please contact the corresponding author directly for reuse.
