# Supplementary figures and images for: The sensor histidine kinase PhcS participates in the regulation of quorum sensing-dependent virulence genes in Ralstonia pseudosolanacearum strain OE1-1
Source: Microbiol Spectr. 2025 Mar 4;13(4):e00059-25. doi: 10.1128/spectrum.00059-25 (PMC11960443; doi:10.1128/spectrum.00059-25)

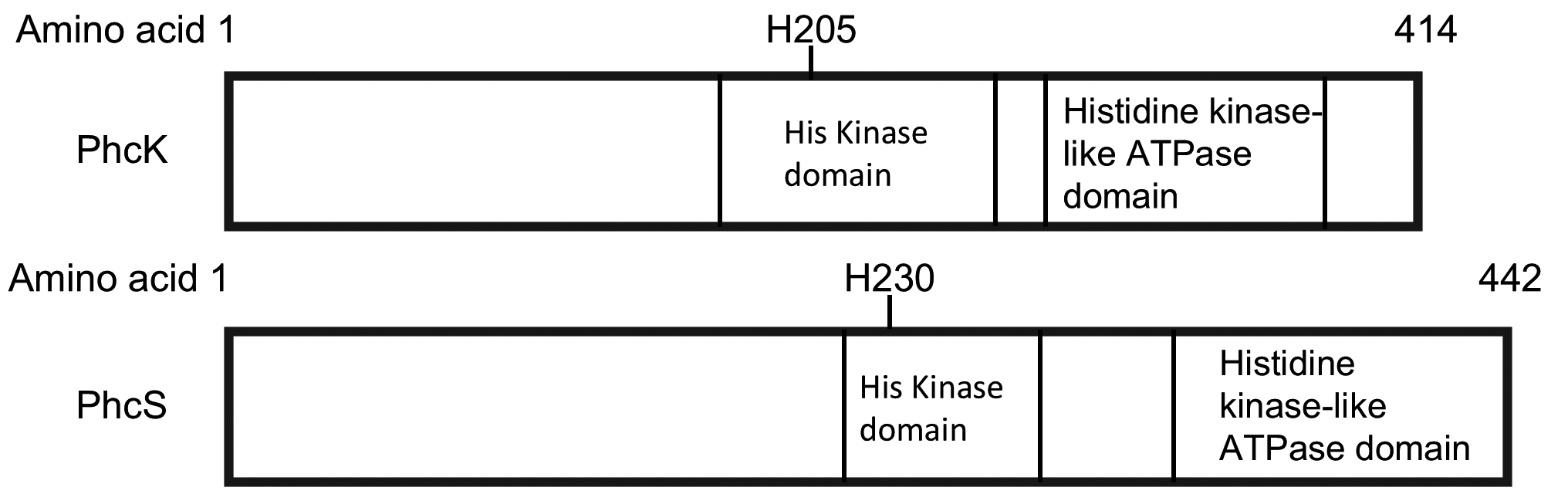

Supplement: Fig S1 — Domains in histidine sensor kinases, PhcK and PhcS, of Ralstonia pseudosolanacearum strain OE1-1 analyzed by the kinasephos2 algorithm (40) using deduced amino acid sequences. [file spectrum.00059-25-s0001.tif]
