# Supplementary material for: The sensor histidine kinase PhcS participates in the regulation of quorum sensing-dependent virulence genes in Ralstonia pseudosolanacearum strain OE1-1
Source: Microbiol Spectr. 2025 Mar 4;13(4):e00059-25. doi: 10.1128/spectrum.00059-25 (PMC11960443; doi:10.1128/spectrum.00059-25)
Supplement: Supplemental material — Fig. S1 legend. [file spectrum.00059-25-s0002.docx]

**Legend of Supplemental Figures**

**FIG S1** Domains in histidine sensor kinases, PhcK and PhcS, of *Ralstonia pseudosolanacearum* strain OE1-1 analysed by the kinasephos2 algorithm (40) using deduced amino acid sequences. His-PhcK205 and His-PhcS230, phosphorylation sites.
